# Supplementary material for: RalGPS2 Interacts with Akt and PDK1 Promoting Tunneling Nanotubes Formation in Bladder Cancer and Kidney Cells Microenvironment
Source: Cancers (Basel). 2021 Dec 16;13(24):6330. doi: 10.3390/cancers13246330 (PMC8699646; doi:10.3390/cancers13246330)
Supplement: Supplementary file 1 [file cancers-13-06330-s001.zip › cancers-1459490-supplementary.pdf]

cancers

Supplementary Materials: RalGPS2 Interacts with Akt and PDK1 Promoting Tunneling Nanotubes Formation in Bladder Cancer and Kidney Cells Microenvironment

Alessia D'Aloia, Edoardo Arrigoni, Barbara Costa, Giovanna Berruti, Enzo Martegani, Elena Sacco and Michela Ceriani

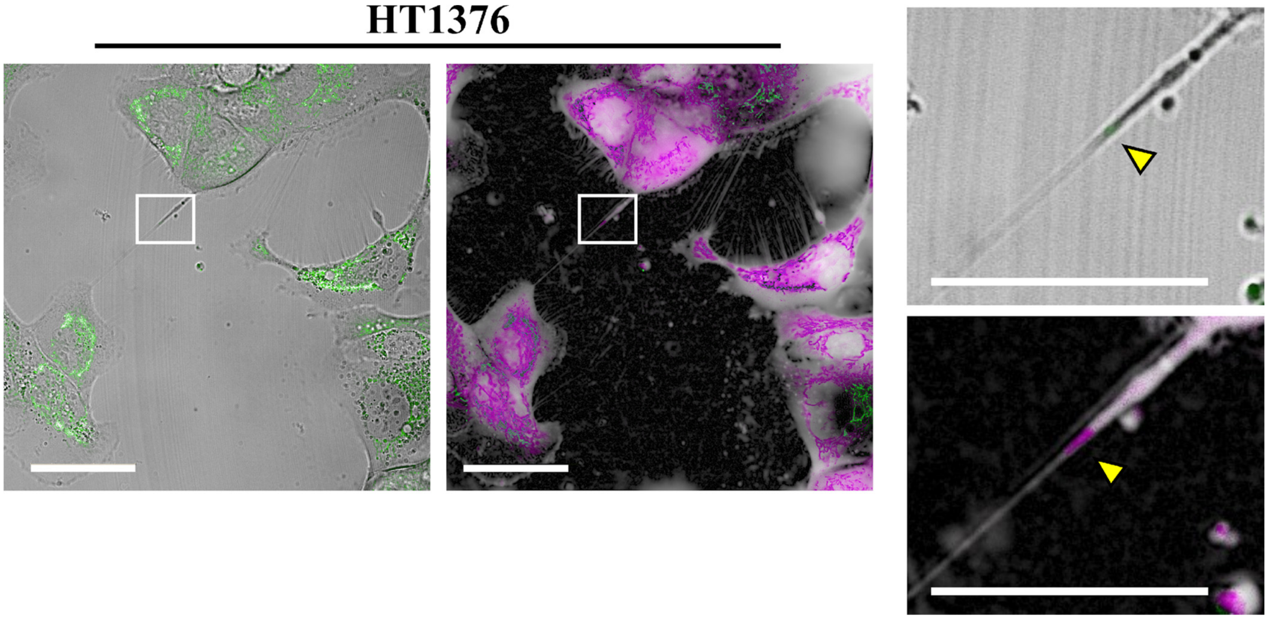

Figure S1. Mitochondria transfer via TNTs in bladder cancer cell lines. HT1376 cells were plated at a density of 1 × 10^4 cells/well on Cell Imaging 24-well Plates. After 24 h, cells were stained with MitoTracker™ Green and live cell images were acquired using Operetta CLS™ equipped with 63x immersion objective in brightfield (left), Digital Phase Contrast (DPC) (right) and fluorescence to detect MitoTracker. Scale bar: 20μm. Yellow triangles indicate mitochondria. Magnified views of mitochondria inside nanotubes are shown.

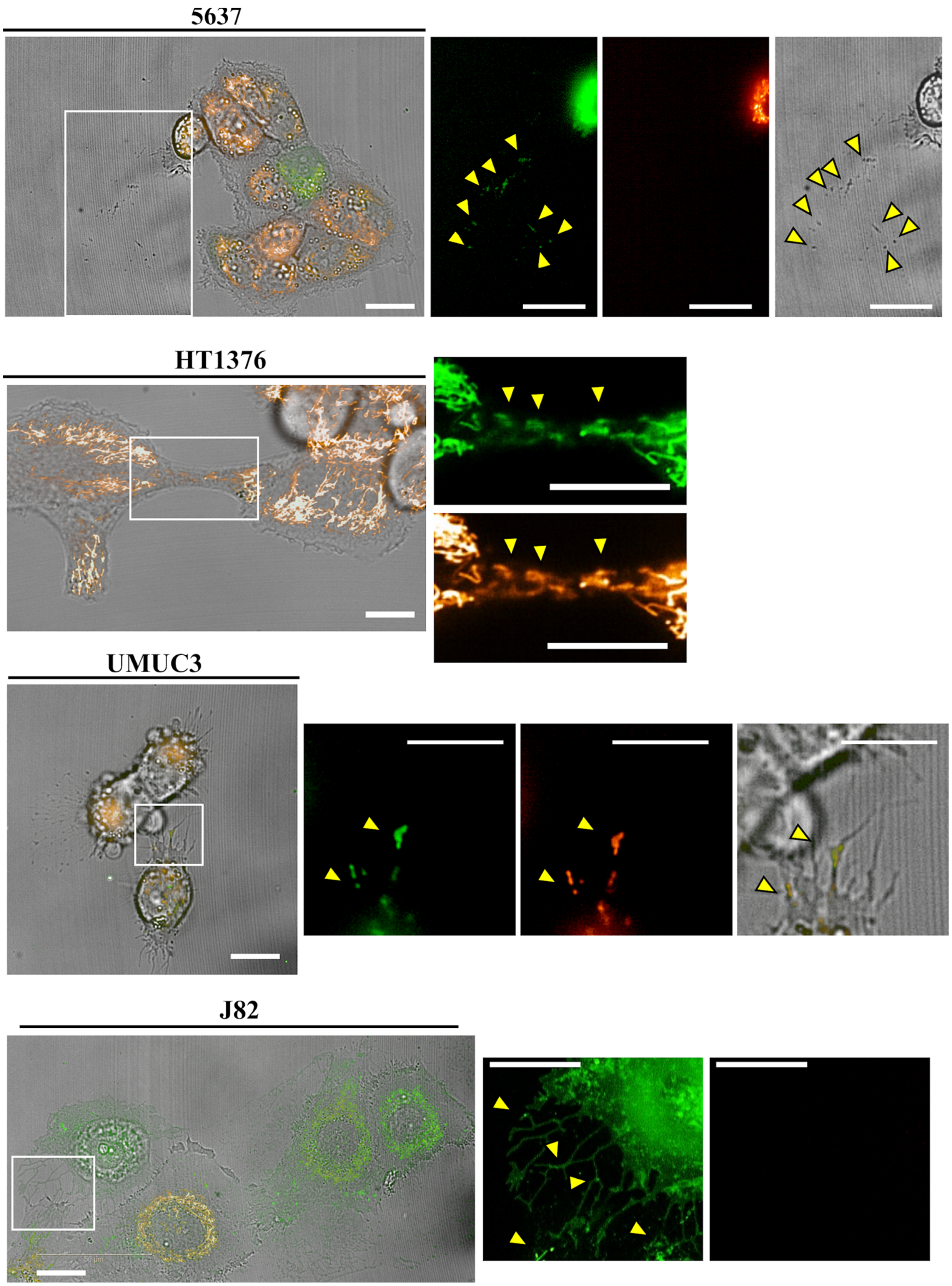

Figure S2. TNTs transfer mitochondria independently by their membrane potential in bladder cancer cell lines. 5637, HT1376, UMUC-3 and J82 cells were plated at a density of 1 × 10^4 cells/well on Cell Imaging 24-well Plates. After 24 h, cells were stained with MitoTracker™ Green together with TMRE (red) and live cell images were acquired using Operetta CLS™ equipped with 63x immersion objective in brightfield (left) and fluorescence to dyes (right). Scale bar: 20µm. Yellow triangles indicate mitochondria. Magnified views of mitochondria inside nanotubes are shown.

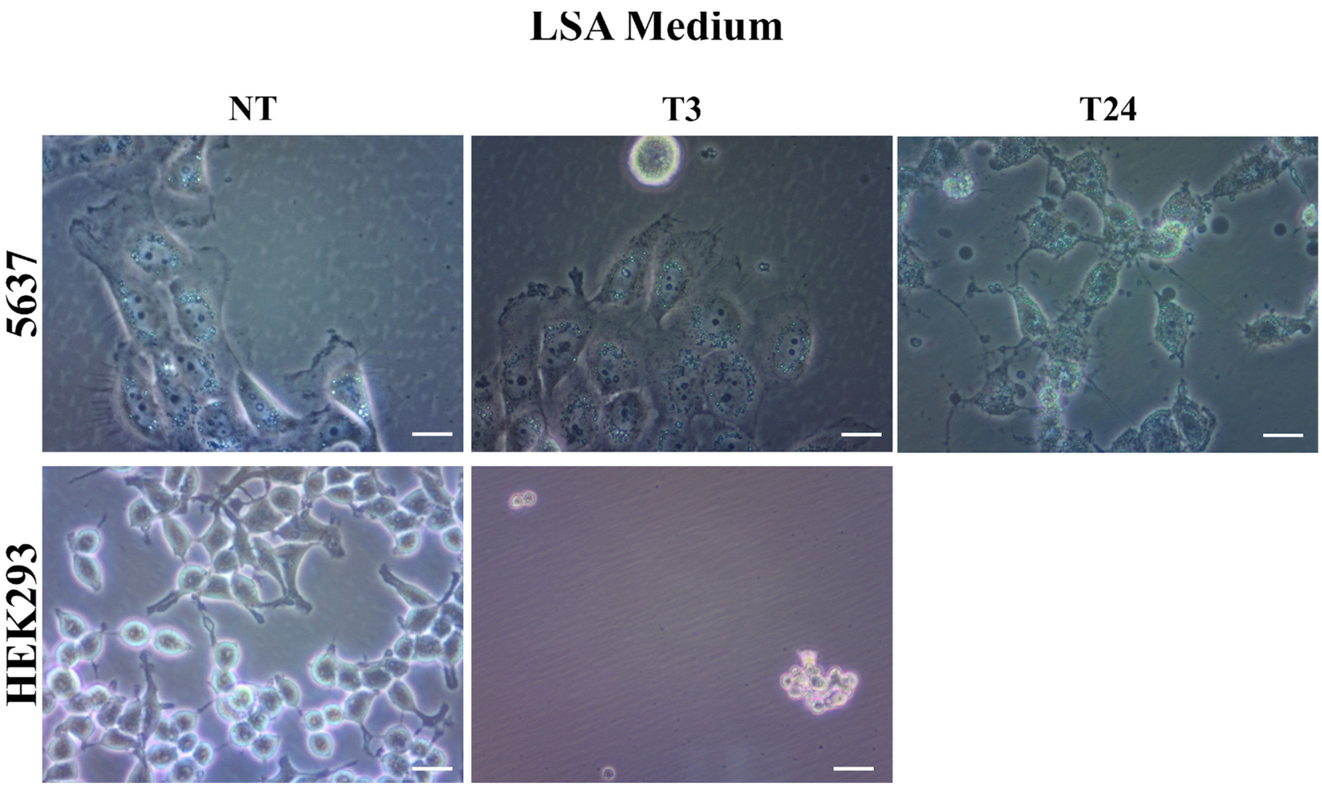

Figure S3. Acidified hyperglycemic low-serum medium induces TNTs formation in 5637 cells. 5637 and HEK293 cells were plated and treated or not treated (NT) with LSA medium for 3 (T3) and 24 hours (T24). Live cells were examined using 20× objective lens on an inverted Olympus CKX41 microscope at different time points. Scale bar: 20μm. NT = not treated; LSA (50 mM glucose, 2.5% FBS, pH 6.6) = Low Serum Acidified.

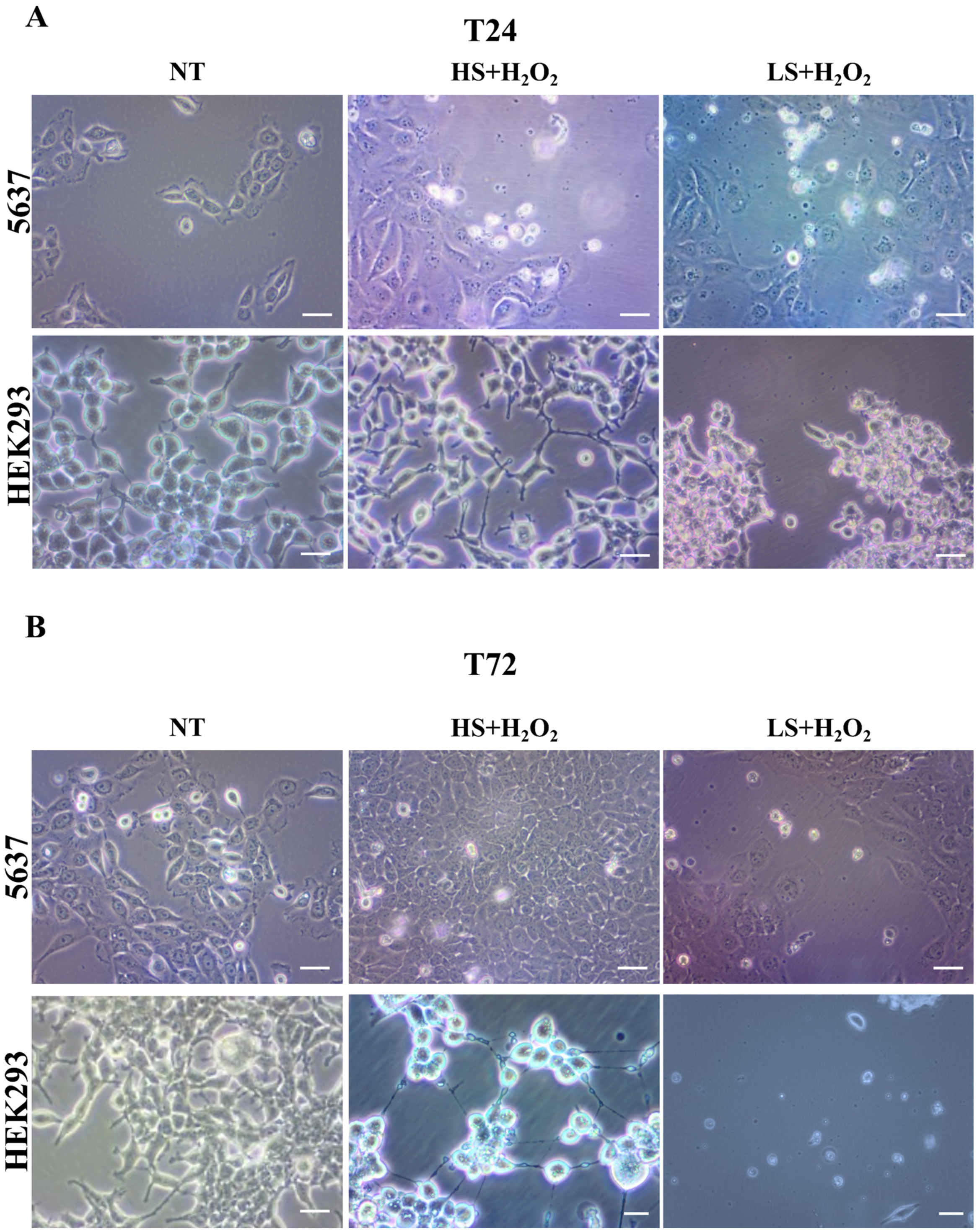

Figure S4. Oxidative stress induces TNTs formation in HEK293 cells. 5637 and HEK293 cells were plated and treated with HS+H_2O_2 or LS+H_2O_2 media. Live cells were examined using 20× objective lens on an inverted Olympus CKX41 microscope at (A) 24h (T24) or (B) 72h (T72) after treatment. Scale bar: 20µm. NT = not treated; HS+H_2O_2 (25 mM glucose, 10% FBS, pH 7.4, 100µM H_2O_2) = High Serum medium with hydrogen peroxide; LS+H2O2 (25 mM glucose, 2.5% FBS, pH 7.4, 100 µM H_2O_2) = Low Serum medium with hydrogen peroxide.

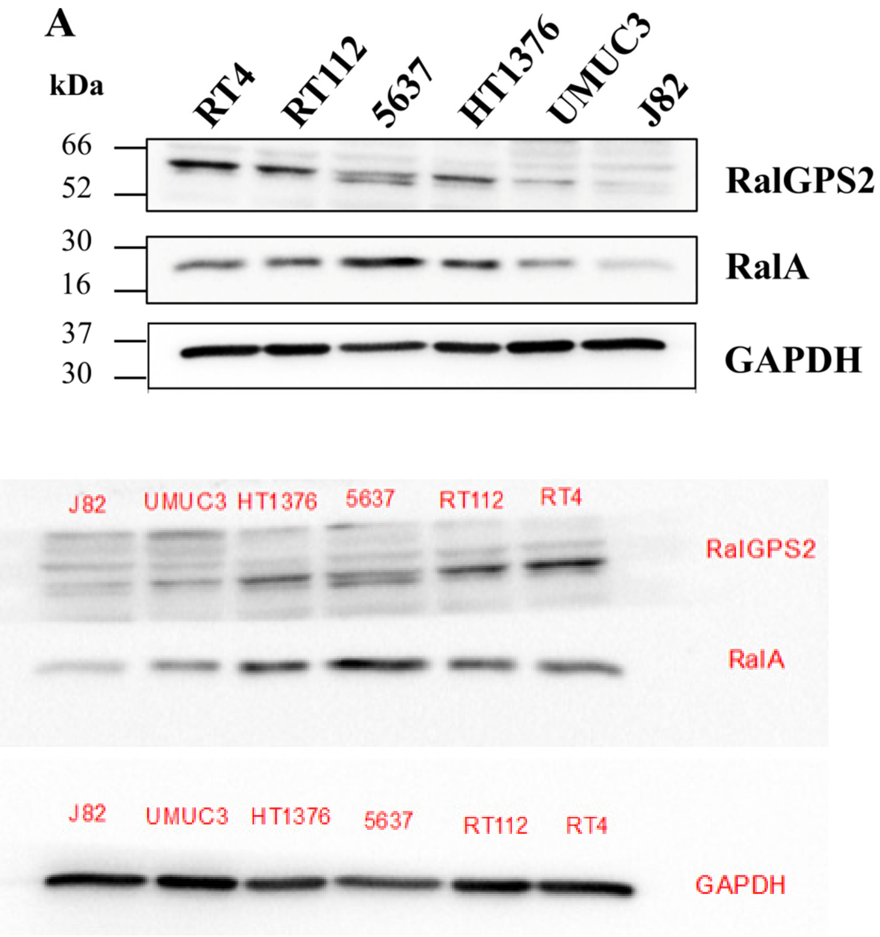

Figure S5. RalGPS2 and RalA expression in bladder cancer cell lines. RT4, RT112, 5637, HT1376, UMUC-3 and J82 cell lysates were separated on SDS-PAGE and blotted to nitrocellulose membrane; blots were probed with anti-RalA or anti RalGPS2 or anti-GAPDH antibodies. GAPDH was used as a loading control. Panel (**A**): representative Western blot results are shown. Panel (**B**): histograms relative to the quantification of RalGPS2 bands. Panel (**C**): histograms relative to the quantification of RalA bands. Data are expressed as mean ± S.E.M. from three independent experiments. Differences among groups were analyzed using a one-way analysis of variance (ANOVA) followed by Tukey's post hoc test. * $p < 0.05$, ** $p < 0.01$, *** $p < 0.001$, **** $p < 0.0001$.

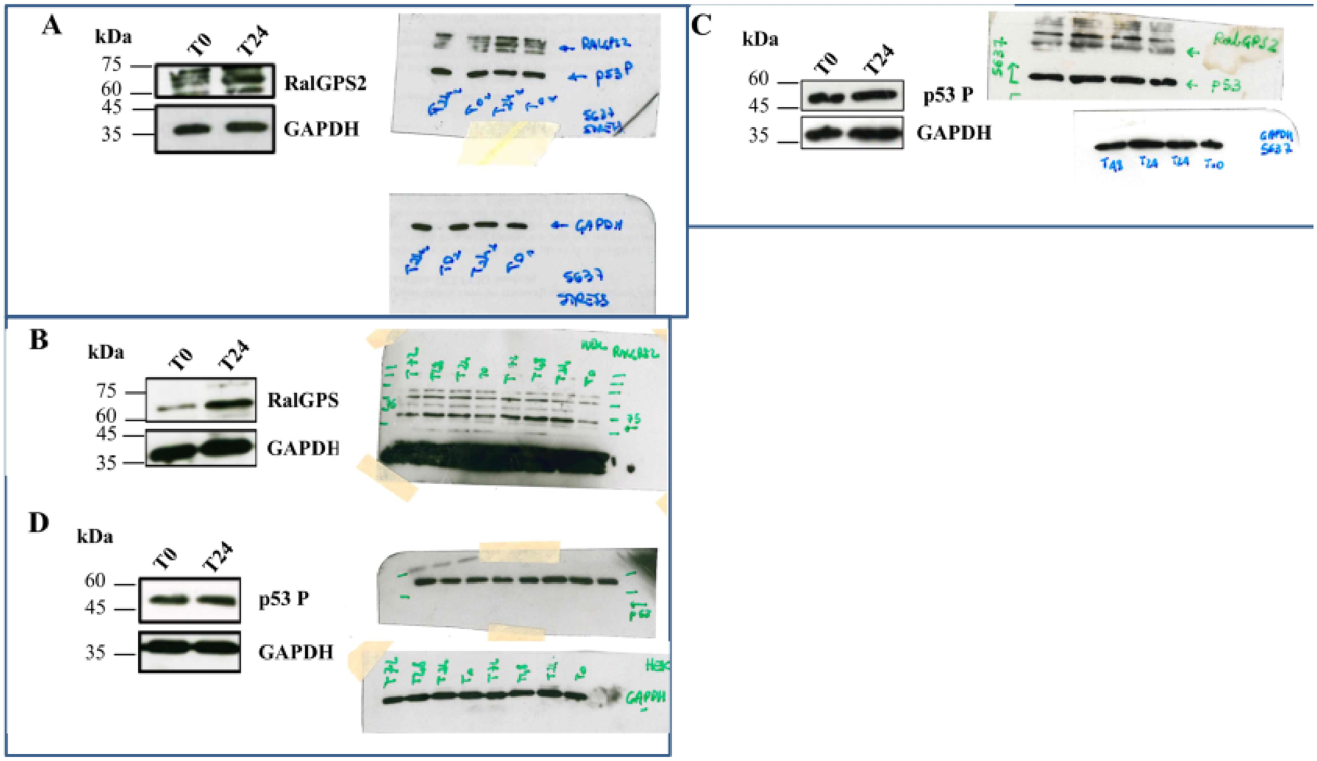

Figure S6. Stress conditions boost RalGPS2 expression in 5637 and HEK293 cells. 5637 and HEK293 cell lysates were separated on SDS-PAGE and blotted to nitrocellulose membrane; blots were probed with anti-RalGPS2 or anti-phospho p53 (p53 P) or anti-GAPDH antibodies. GAPDH was used to normalize sample loading. Panels (**A**,**C**): expression of RalGPS2 and p53 P in 5637 cells treated with LSA medium for 24 h. The panels show representative western blot results and the quantification of (**A**) RalGPS2 or (**C**) p53 P expression. Panels (**B**,**D**): expression of RalGPS2 and p53 P in HEK293 cells treated with HS+H_2O_2 medium for 24 h. The panels show representative western blot results and the graphical representation of (**B**) RalGPS2 or (**D**) p53 P expression. Data are expressed as mean ± S.E.M. from three independent experiments. Differences among groups were analyzed using Student's *t*-test. ** $p < 0.01$.

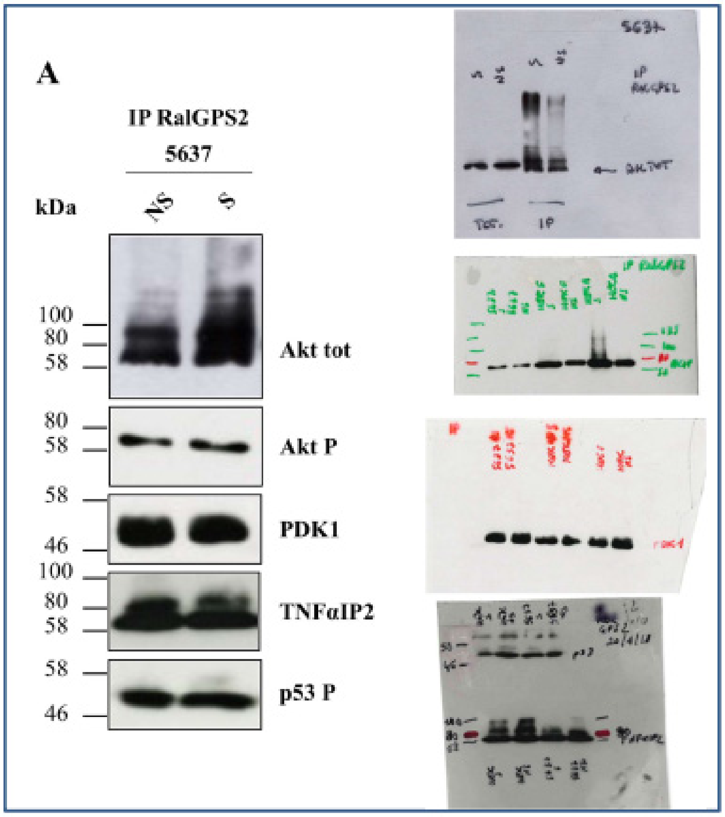

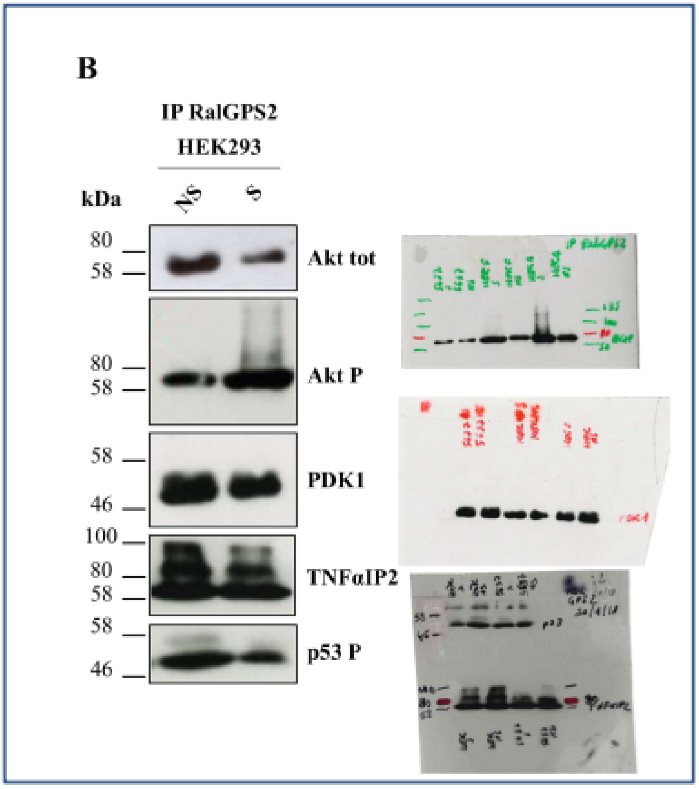

Figure S7. RalGPS2 interacts with Akt, PDK1, TNFαIP2 and p53 P in 5637 and HEK293 cells. (**A**) 5637 and (**B**) HEK293 cells were plated in 100 mm dishes and the day after were first serum-starved for 18 h and then stimulated with 10% FBS

for 15 min (S) or left unstimulated (NS). Subsequently, immunoprecipitation (IP) with anti-RalGPS2 antibodies was performed, as indicated in each panel. Immunoprecipitates were assessed for the presence of total Akt (Akt tot), phospho-Akt (Akt-P), PDK1, TNFαIP2 and phospho-p53 (p53 P). Three independent experiments were performed.

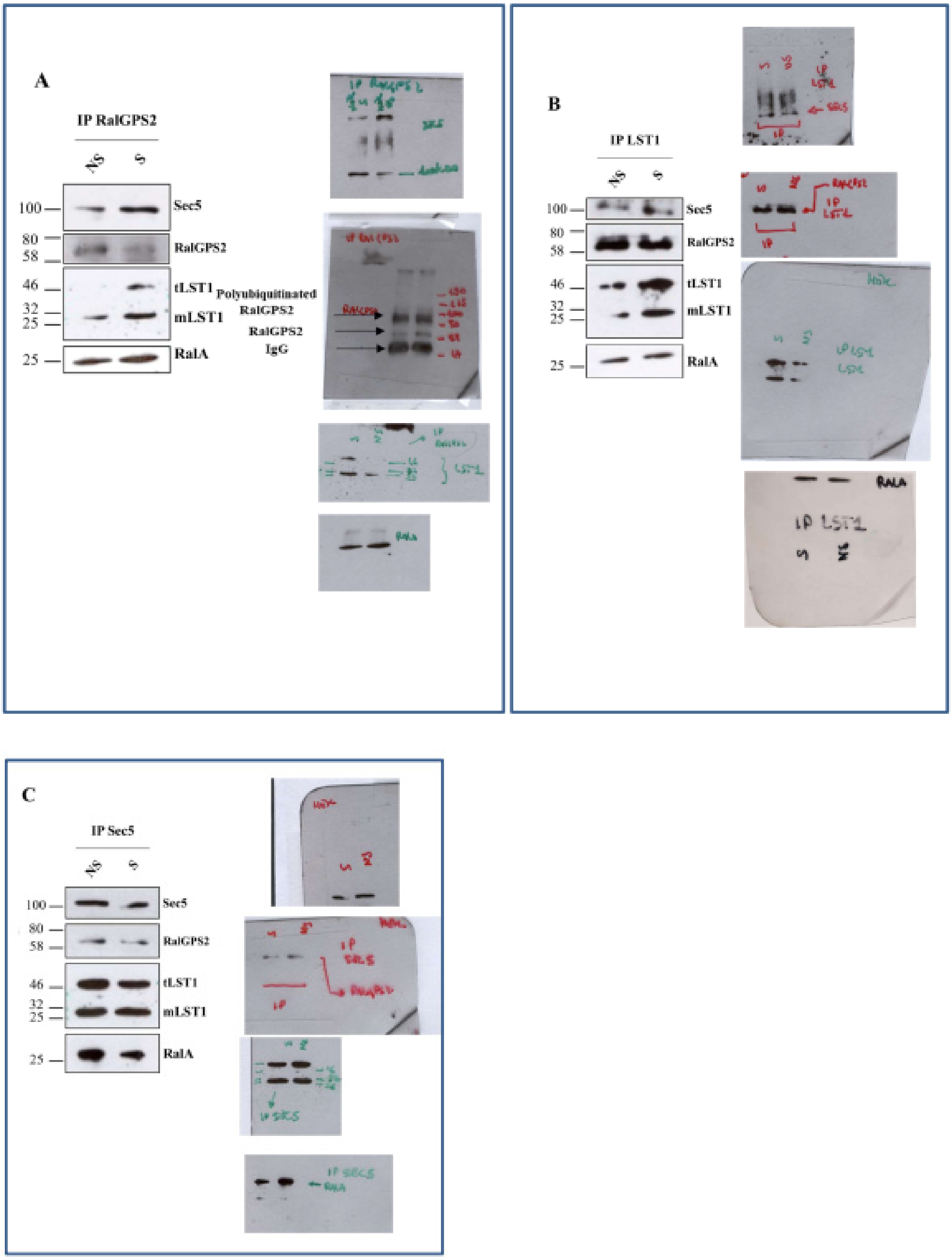

Figure S8. RalGPS2 interacts with RalA, LST1 and Sec5 in HEK293 cells. HEK293 cells were plated in 100 mm dishes, and the day after, were first serum-starved for 18 h and then stimulated with 10% FBS for 15 min (S) or left unstimulated (NS). Subsequently, immunoprecipitation (IP) with (**A**) anti-RalGPS2, (**B**) anti-LST1 or (**C**) anti-Sec5 antibodies were performed, as indicated in each panel. Immunoprecipitates were probed for Sec5, LST1, RalA and RalGPS2 presence. tLST1: LST1 trimerous; mLST1: LST1 monomer.
